# Study protocol of a randomized controlled trial evaluating home treatment with peer support for acute mental health crises (HoPe)

**DOI:** 10.1186/s12888-022-04247-w

**Published:** 2022-09-19

**Authors:** Britta Reinke, Candelaria Mahlke, Christina Botros, Alexa Kläring, Martin Lambert, Anne Karow, Jürgen Gallinat, Antonia Zapf, Ann-Kathrin Ozga, Alexandra Höller, Nadia Bustami, Jens Reimer, Jenny Lüdtke, Oliver Schaper, Martin Lison, Andreas Bechdolf, Johanna Baumgardt, Jennifer Spiegel, Olaf Hardt, Sandeep Rout, Sonja Memarzadeh, Sebastian von Peter, Julian Schwarz, Claudia Langer, Sabine Glotz, Karel Frasch, Nicolas Rüsch, Ulf Künstler, Thomas Bock, Thomas Becker

**Affiliations:** 1 Department of Psychiatry and Psychotherapy, University Medical Center Hamburg-Eppendorf, Hamburg, Germany; 2 Department of Medical Biometry and Epidemiology, University Medical Center Hamburg-Eppendorf, Hamburg, Germany; 3 Department of Psychiatry and Psychotherapy, Psychiatric Hospital Lüneburg, Lüneburg, Germany; 4 Department of Psychiatry and Psychotherapy, Gesundheit Nord - Bremen Hospital Group, Bremen, Germany; 5 Department of Psychiatry, Psychotherapy and Psychosomatic Medicine, Vivantes Hospital Am Urban Und Vivantes Hospital Im Friedrichshain, Charité – Universitätsmedizin Berlin, Berlin, Germany; 6 Department of Psychiatry, Psychotherapy and Psychosomatics, Vivantes Friedrichshain Hospital, Berlin, Germany; 7 Department of Psychiatry and Psychotherapy, University Hospital Cologne, Cologne, Germany; 8 Department of Psychiatry, Psychotherapy and Psychosomatics, Vivantes Neukölln Hospital, Berlin, Germany; 9 Department of Psychiatry and Psychotherapy, Brandenburg Medical School Theodor Fontane, Immanuel Clinic Rüdersdorf, Rüdersdorf, Germany; 10 Department of Psychiatry II, Ulm University, Günzburg Regional Hospital, Günzburg, Germany; 11 Donauwörth Regional Hospital, Donauwörth, Germany; 12 Department of Psychiatry and Psychotherapy, Asklepios Western Hospital Hamburg, Rissen, Germany

**Keywords:** Home treatment, Peer support, Severe mental illness, Randomized controlled trial, Mental health care, Crisis resolution teams

## Abstract

**Background:**

Home treatment (HT) is a treatment modality for patients with severe mental illness (SMI) in acute mental crises. It is frequently considered equivalent to psychiatric inpatient treatment in terms of treatment outcome. Peer Support (PS) means that people with lived experience of a mental illness are trained to support others on their way towards recovery. While PS is growing in international importance and despite a growing number of studies supporting its benefits, it is still not comprehensively implemented into routine care. The HoPe (Home Treatment with Peer Support) study investigates a combination of both – HT and PS – to provide further evidence for a recovery-oriented treatment of psychiatric patients.

**Methods:**

In our randomized controlled trial (RCT), HT with PS is compared with HT without PS within a network of eight psychiatric clinical centers from the North, South and East of Germany. We investigate the effects of a combination of both approaches with respect to the prevention of relapse/recurrence defined as first hospitalization after randomization (primary outcome), disease severity, general functioning, self-efficacy, psychosocial health, stigma resistance, recovery support, and service satisfaction (secondary outcomes). A sample of 286 patients will be assessed at baseline after admission to HT care (data point t_0_) and randomized into the intervention (HT + PS) and control arm (HT). Follow-Up assessments will be conducted 2, 6 and 12 months after admission (resulting in three further data points, t_1_ to t_3_) and will be analyzed via intention-to-treat approach.

**Discussion:**

This study may determine the positive effects of PS added to HT, prove additional evidence for the efficacy of PS and thereby facilitate its further implementation into psychiatric settings. The aim is to improve quality of mental health care and patients’ recovery as well as to reduce the risk of relapses and hospitalizations for patients with SMI.

**Trial registration:**

The trial is registered with ClinicalTrials.gov: NCT04336527, April 7, 2020.

## Background

There are two innovative approaches in mental health care with a growing importance for especially treatment of patients with severe mental illness (SMI): Home Treatment (HT) – acute psychiatric care at home – and Peer Support (PS) – accompanying patients by individuals with lived experience in mental health crisis.

HT is a care model providing an alternative to acute inpatient stays in clinical settings as mobile teams visit patients in their home setting, internationally also referred to as crisis resolution teams (CRT) [1, 2]. Patients with SMI make up the main population receiving HT interventions [3]. In Germany, in addition to regional pilot projects [4], HT was recently implemented into regular psychiatric care as Inpatient Equivalent Home Treatment (IEHT) by law (PsychVVG, technical term coined by the Federal Ministry of Health: *Stationsäquivalente Behandlung, STÄB* – agreed on Jan 1, 2017, entered into force as of Jan 1, 2018; [5]). The operationalized HT model varies and surveys have revealed divergence in organization and implementation [6, 7], resources for home treatment (HT), and models of service delivery [8]. There have been some inconsistent findings regarding the efficacy of HT and the magnitude of changes in outcome, e.g. in reducing admission and days in admission [9–11]. Furthermore, the number of randomized controlled trials (RCT) investigating HT is limited. Nonetheless, a growing number of studies supports its positive effects: The 2009 National Institute for Health and Care Excellence (NICE) pooled reanalysis of clinical guidelines for Schizophrenia [12], which included 883 participants and six RCTs, found strong evidence for the reduction of admissions, shorter treatment duration, and fewer incidents of dropout while being treated, as well as increased satisfaction and cost-effectiveness, compared with standard care (admission to psychiatric wards). The latest Cochrane review of crisis intervention for patients with SMI also included two RCTs (mobile crisis teams, crisis units based in home-like residential houses). It showed a reduction of family burden and improvements of mental and global state, compared to standard care [13]*.* Given specific HT characteristics and the importance of model fidelity, a systematic review including 16 studies of different methodologies (RCTs as well as natural experimental studies) also indicates that HT can reduce hospital admission and inpatient bed days and increase patients’ satisfaction, compared to standard care [14]. These positive results are in line with other German studies either showing HT comparably effective as inpatient treatment [15, 16] or favoring HT against inpatient treatment concerning risk of hospital readmission and length of stay [17], depressive symptom severity, clinical and functional impairment and cost-effectiveness [18], service disengagement, psychopathology, psychosocial functioning, quality of life, satisfaction with care, adherence, and involuntary admissions [19]. A recent cluster-randomized trial proved even lower inpatient admissions and bed-use as well as an increase of staff psychological health after a one-year improvement program for crisis resolution teams [20, 21].

In a naturalistic study conducted in 17 German care networks providing HT, an increase of patients’ psychosocial functioning was related to staff experience and staff’s effort invested [22]. In a systematic review with 13 papers regarding service users’ experiences with HT besides ‘access and availability’ and ‘dealing with crisis in an everyday life contact’ the fact of ‘being understood as “normal” human beings’ was identified as key point of HT interventions [23]. These might be factors specific to PS.

PS workers use their personal background in mental health crises to support patients. In Germany, they can complete e.g. an Experienced Involvement (EX-IN) training of PS education [24] and contribute to mental health care by sharing their experiences and setting an example for recovery. Internationally various forms of PS are implemented in different countries [25].

Reviews on PS report few RCTs [26–28] in a wide range of different PS service settings. Results show that PS encourages hope, empowerment and recovery [27], and quality of life in patients with SMI [26]. Furthermore, there was a small difference in crisis and emergency service use as well as in meeting clients’ needs favoring the involvement of peers [28]. Further reviews also including non-randomized and some more recent controlled trials [29, 30] show significant differences in patient activation, hope, empowerment, and self-efficacy. Single RCTs show that peer-led groups positively affect internalized stigma [31], illness-management and adherence [32], and users’ social networks and support [33]. Whereas some reviews did not find remarkable differences in clinical outcomes like mental health symptoms or service use and hospitalization [27, 28], a recent RCT showed a reduced rate of hospital readmissions in a group that received peer supported interventions compared to a control condition [34]. Because the number of studies is limited and quality of evidence is partially low [35], high quality RCTs with clearly defined interventions, sufficient peer training, large sample sizes, adequate randomization, blinding of measurements, and the use of recovery outcomes with longer follow-up periods are called for [27, 28, 36]. Furthermore, an important aspect is to evaluate the fidelity of PS interventions and the principles underlying the role of PS and the positive effects of PS work [37].

### Objectives

Although both treatment options – HT and PS – are explicitly recommended in the German guidelines on psychosocial therapies regarding SMI [38], they are still considered innovative, are not comprehensively implemented across Germany [39] and are seldom combined. Following the successful implementation of peer projects in Germany, e.g. at the University Medical Centre Hamburg-Eppendorf [40], the effects of PS added to HT deserve evaluation, given PS might strengthen HT. The aim of the present study is to investigate the efficacy of the combination of both approaches in acute care – a HT plus PS intervention versus HT alone – to improve mental health care for patients with SMI.

### Hypotheses

It is hypothesized that a peer-supported home-delivered treatment (HT plus PS) is more efficacious than a professional-led home-delivered treatment (HT alone) with respect to the time until (first) hospital readmission (primary outcome), self-efficacy, psychosocial health, recovery orientation, stigma-resistance, and service satisfaction. Furthermore, it is hypothesized, that a peer-supported home delivered treatment (HT plus PS) is as effective as a professional-led home-delivered treatment (HT alone) with respect to disease severity and general functioning.

### Design

The trial is designed as a prospective open multicenter parallel group RCT to test the efficacy of HT with PS (intervention group) against sole HT (control group) at eight different study sites throughout Germany. Patients admitted to HT care are randomly allocated to the intervention and control group in a 1:1 ratio (block randomization stratified by study site). All participants will provide written informed consent before inclusion in the study.

## Methods

### Study setting

Data collection takes place at eight psychiatric hospitals, both academic and community clinics, in both rural and urban environments in the North, East, and South of Germany. All settings offer HT as regular care for psychiatric patients. In most cases, PS workers are already part of the HT teams and there has been a longstanding cooperation between HT teams and external PS workers. In some cases (additional) PS workers were employed within the HT team during study preparation. All study sites are experienced in working with PS workers. The location of all study sites can be obtained from the affiliations.

Data collection starts individually at the participating hospitals due to individual organizational prerequisites as well as delays caused by the current COVID-19 pandemic. Each study site will recruit patients for 12 months which are followed for at least 12 months (with the last individual measurement point t_3_ for secondary outcomes 12 months after t_0_, i.e. admission). For the primary outcome all patients are followed until one year after the last patient was included in the study at a study site. An extension of the recruitment period for each study site for up to 6 months for delays in patient acquisition due to COVID-19 restrictions and concerns (hospital closures, reluctant patients, etc.) was granted to ensure sufficiently large sample sizes. The first participating hospital started recruitment in July 2020, the last hospital is expected to start data collection at the latest in October 2021.

### Eligibility criteria

Care providers administering the intervention include subjects with a minimum age of 18 years, a diagnosis of an SMI (defined by diagnoses of ICD-10: F2, F3, F6, F41, F42), sufficient German language skills, and the indication of hospital admission because of an acute mental health condition. Exclusion criteria are a primary diagnosis of organic or substance use disorders (ICD-10: F0 or F1 [41]), acute suicidality, and a verbal or cognitive impairment severe enough to be unable to give written informed consent.

### Interventions

Patients in the *intervention group* receive a combination of HT by a multi-professional team with PS: PS workers are involved during the whole treatment duration. After discharge a maximum of five additional contacts are possible. Contacts to a PS worker take place at least one time per week in 1:1 contacts or in combination with other members of the HT team. Important aspects of PS contacts are defined as focus on personal resources, support of self-determination and autonomy, practical support in daily tasks, recover- and need-orientation, building bridges between patients and care providers, and trialogical work with inclusion of relatives or close persons. The so-called ‘trialogue’ format [42] is a term used in Germany to designate discussion meetings involving peers, carers and professionals focusing on the experience of, coping with, and recovery from mental health problems with special attention to the peer, carer and professional perspectives, contrasts, differences and perhaps similarities in experience. As far as possible (e.g. due to limited working hours), PS workers are included into all relevant treatment decisions. To ensure a standardization of treatment, an intervention guideline was established together with PS workers. Furthermore, there was an online training for PS workers regarding relevant aspects of peer work and the peer role within the intervention. All eligible PS workers had to have completed a standardized PS training, e.g. *Ex-In (Experienced Involvement)* [24] or *Honest, Open, Proud* [31, 43].

The *control group* receives conventional HT by a multi-professional team without contacts to a PS worker (treatment as usual). Multi-professional teams consist of nursing staff, psychiatrists/physicians, occupational therapists, social workers, and psychologists/psychotherapists. They provide acute mental health treatment by mobile care teams within the patients’ home setting or familiar environment that is equivalent to inpatient treatment (i.e. crisis intervention, supportive and therapeutic conversations, pharmacotherapy, support in daily life and problem solving, transfer to outpatient care and support [1, 44].

The duration of treatment depends on the individual clinical indication and may vary between two and several weeks, equivalent to inpatient stays. In case of readmission within the first six months after study enrolment patients receive the same intervention as during their first treatment – after this period of time they receive treatment as usual. Patients within the control group do not receive any PS by caregivers during the whole study period.

Fidelity of treatment is measured by use of a fidelity scale examining adherence to the intervention protocol in line with Lloyd-Evans and colleagues. [45].

### Outcomes

Primary outcome is *time to first readmission to inpatient hospital treatment* (follow-up monitoring until one year after the last patient was included).

#### Secondary outcomes are:

- Change from baseline in *self-efficacy expectation* mean scores as assessed by the German scale “Skala zur Allgemeinen Selbstwirksamkeitserwartung” [SWE] [46].
- Change from baseline in *psychosocial health* mean scores as assessed by the German scale “Hamburger Module zur Erfassung allgemeiner Aspekte psychosozialer Gesundheit für die therapeutische Praxis” [Health-49] (subscales: somatoform complaints, depressiveness, phobic anxiety, psychological well-being, interactional problems, self-efficacy, activity and participation, social support, social stress [47]).
- Change from baseline in *stigma resistance* mean scores as assessed by the “Internalized Stigma of Mental Illness” [ISMI] scale – subscale stigma resistance [48].
- Change from baseline in *disease severity* mean scores as assessed by the “Clinical Global Impression” [CGI] scale [49].
- Change from baseline in *general functioning* mean scores as assessed by the “Global Assessment of Functioning scale [GAF] [50].
- Change from baseline in recover support as assessed by [INSPIRE] [51].

Times of assessment are at admission, and two, six, and twelve months after admission.

#### Further secondary outcomes are:

- Means in *service satisfaction* scores as assessed by the German version of the Client Satisfaction Questionnaire [ZUF-8] [52].
- Means in *recovery support* scores as assessed by Brief INSPIRE [51].

Both are assessed at two and six months after inclusion into study.

### Participant timeline

**Fig. 1:**
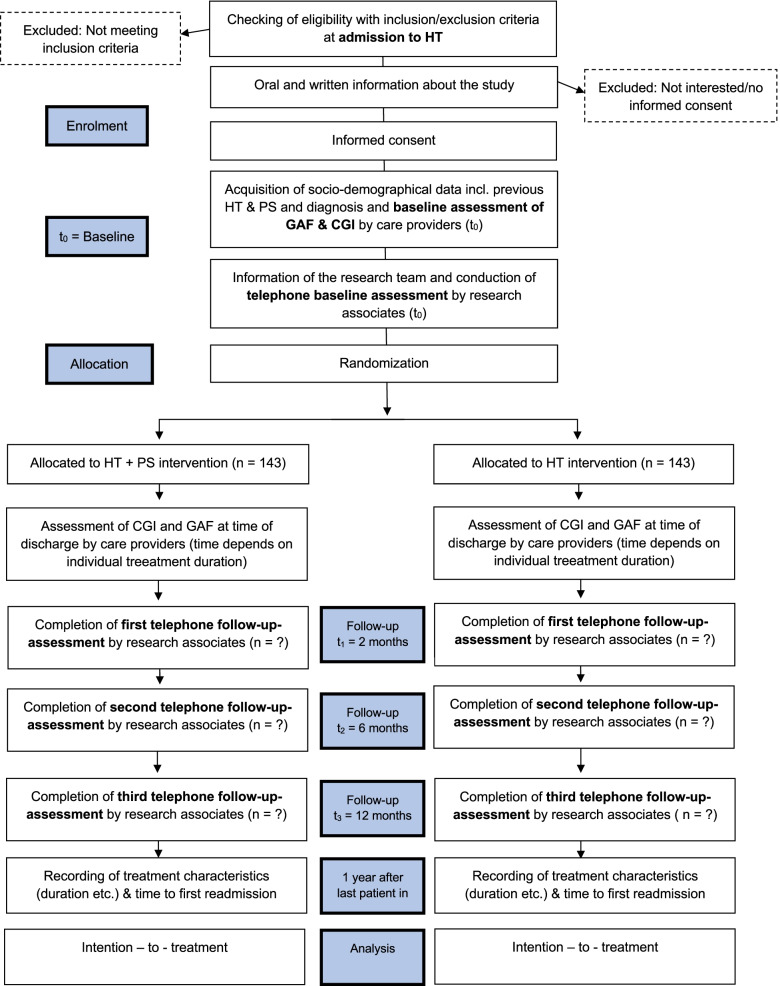
Participant Timeline

The participant timeline is visualized in Fig. 1.

### Sample size

For the purpose of sample size calculation, data for the HT alone group were collected from routine data of University Medical Center of Hamburg-Eppendorf, Department of Psychiatry and Psychotherapy. For the HT plus PS group data was collected from a former in-house study (unpublished routine data). From these data, one-year admission-free survival rates of 58% and 73%, respectively, were calculated corresponding to a hazard ratio of 0.577. To detect a difference between random groups of this order of magnitude with a power of 80%, *N* = 254 patients in total (127 patients per group; total event count = 106) are required if the recruitment takes 12 months and there are at least 12 months follow-up to an administrative censoring. This calculation includes a 30% loss-to-follow up over two years of follow-up time. With an accrual period of 18 months and the same assumption as above a sample size of *N* = 232 (total event count 106) is needed. This latter analysis was only added since recruitment intensity might be low due to COVID-19 restrictions. Additionally, it was checked how many patients would be needed to demonstrate a medium effect size (Cohen’s d = 0.4) with a power of 80% and a two-sided type I error of 5% in the secondary continuous outcomes. This resulted in 100 patients per group (200 patients in total) and with an expected dropout rate of 30%, in 286 patients in total (143 patients per group). Therefore, the total sample size of 286 was chosen. Sample size was calculated with PASS 2008 based on the logrank test (using proportion surviving) [53] and the two-sample t-test using an effect size.

### Recruitment

Patients regularly admitted to HT at one of the eight participating study sites and fulfilling eligibility criteria are informed about the study by care providers at time of admission. In most cases, patients are directly admitted. Nonetheless, it is also possible that they are transferred from inpatient treatment to HT. Patients receive verbal and written information about the study. If study information is not possible due to acute symptom severity, admission to the study can be delayed for up to 14 days until informed consent can be given. Until enrolment to the study, these patients receive standard HT care without involvement of peers. For enrolment, a minimal planned duration of treatment of two (further) weeks or ten home visits is predefined. Before giving written informed consent, patients have the possibility to additionally consult local research teams for patient information. This offer holds for the duration of the entire study. After having given written informed consent, care givers contact the local research team, who is then conducting the first telephone interview (see below). Study participation is voluntary. If negated or in case of dropout, patients receive treatment as usual by the HT team without any harm. The recruitment period continues for up to 18 months at each site to reach an average target sample of 36 participants per site.

### Methods: assignment of interventions

#### Allocation

For allocation to treatment and control group, a randomly permuted block randomization with varying block size stratified by site is used to assign patients in a 1:1 ratio by an online randomization service (www.randomizer.at). Care providers are instructed to log in to an online interface to receive allocation after completing baseline assessment. After completion of the first telephone interview with the local research associates, patients are informed about allocation by HT care providers at their subsequent visit.

#### Blinding

HT care providers and patients are blinded at time of baseline ratings (GAF; CGI). Randomization and allocation to treatment are done after baseline rating. For compelling reasons, HT care providers’ second rating of GAF and CGI at time of discharge is not blinded nor are patients at further times of data acquisition because they will have received intervention. Research associates accomplishing telephone interviews are blinded whenever possible. That is, at all four measurement points, namely at admission (t_0_), 2 months after admission (t_1_), 6 months after admission (t_2_), and 12 months after admission (t_3_). In telephone interviews at t_1_, t_2_, and t_3_ patients are explicitly instructed not to reveal any information about their group allocation against research associates to ensure blinding of outcome assessment. The statistical analysis will be conducted in a blinded manner.

### Methods: data collection, management, analysis

#### Data collection methods

Data are collected at baseline by HT staff and research associates, at time of discharge by HT staff, and at the three follow-ups (at 2, 6 and 12 months) by local research teams.

#### Primary outcome

Data on *time to first readmission to inpatient hospital treatment* is collected from the respective hospital IT database. According to the intention-to-treat analysis, primary outcomes of all participants are collected – also in case of dropout from treatment or follow-up assessment.

#### Secondary outcomes

The main part of data collection of secondary outcomes is carried out by research associates using four telephone interviews lasting between 30 to 45 min. They take place at time of enrolment and at a 2-months, 6-months, and 12-months follow-up. To provide a standardized procedure for research associates, a guideline for instruction of telephone interviews was prepared and trained. To facilitate oral self-ratings for participants, visual response scales are handed out in advance.

At time of enrolment, *socio-demographical data* (age, gender, domestic and occupational circumstances) and *information about previous HT and PS* and *current diagnoses* of participants are collected by local HT care providers, as well as ratings of CGI and GAF. HT Care providers are trained in GAF ratings if not used to it or GAF ratings had not been implemented to clinical routine before. At the end of the treatment, care providers again rate CGI and GAF. Additionally, it is registered *how treatment was concluded* (regular discharge/ termination of treatment by clinicians/ drop-out by patient/ in-house admission) and if there was a *change in diagnosis*. Furthermore, *days of treatment, number of treatment contacts, and duration of treatment contacts* are recorded for each participant. For patients having received PS, *treatment day of first PS contact, number of PS contacts* (1:1, within the HT team, in a group setting*), number of aftercare PS contacts* and *duration of PS contacts* are additionally recorded.

#### Safety outcomes

Safety outcomes (i.e. adverse events, AEs) will be recorded as part of the regular documentation. As such, we do not expect major adverse events, especially none caused by the intervention (peer support). However, in terms of assessment of safety and harms, we will record and report dropout from study, diagnosis pre/post, type of discharge from treatment (as planned/early termination by patient or team/admission to inpatient treatment), severity of mental disorder (CGI and GAF, pre/post), hospital admissions due to somatic diseases, and death.

#### Data management

After having given written informed consent, each participant receives a subject ID stored in a locked key list. All data acquired will be stored and locked solely with this ID that does not contain any patient identifying information. Data will be stored according to a data management plan ensuring data security.

Data entry will be prepared by the coordinating study site at the UKE Hamburg by establishing a common template for data input from telephone interviews. Where possible, the research associates at each study site will enter their acquired data directly, otherwise this will be done centrally. The paper version of the interviews will be stored until all final check-ups have been conducted.

### Statistical methods

Patients will be described with respect to relevant baseline characteristics, both overall and separately for the two randomized groups. Categorical data will be summarized by numbers and percentages. Continuous data will be summarized by mean, standard deviation, median, inter-quartile-range, and range. The number of available observations and the number of missing observations will be presented separately for the treatment groups. Tests of statistical significance will not be undertaken for baseline characteristics; rather the clinical importance of any imbalance will be noted [54].

The intention-to-treat analyses of primary data will be based on the available clinical data from all randomized patients. Only the primary analysis will be treated in a confirmatory manner. The aim is to show a difference between the peer-supported HT group compared to the professional-led HT group with respect to the primary endpoint. For the primary endpoint, a stratified logrank test for a comparison of the treatment groups with strata defined by study center will be performed. The two-sided type I error will be set at 5%. A stratified (stratum = study center) Cox proportional hazard model will be applied to estimate a treatment effect (hazard ratio). The hazard ratio will be reported along with the corresponding 95% confidence interval. An additional analysis will be conducted on a per-protocol (PP) analysis set. The PP population consists of the complete cases without severe protocol violations. Severe protocol violations are defined according to the intervention protocol, e.g. too few peer contacts in the intervention group (HT plus PS). Extended analyses (ITT-population) with inclusion of several covariates will be performed using the stratified Cox model. In particular, patient sex will be included as a covariate, and an interaction term sex*intervention will be added to the model for subgroup analysis. Besides patient sex, further covariates/subgroups include patient age, main diagnosis, composition of household (living alone/with partner/with kids etc.), occupational situation, severity of mental disorder (according to CGI and GAF) at admission, earlier exposure to peer support (j/n), and location of psychiatric clinical center (North/South/East and rural/urban).

The secondary endpoints will be examined in an exploratory manner with linear mixed models. In case of skewed distributions, transformations will be considered (e.g. log-transformation). In case of zero-inflated data, mixed zero-inflated models or negative binomial models will be used.

Safety outcomes (i.e. adverse events, AEs) will be determined by group using frequency tables and if possible using logistic regressions to compare the event frequencies. Interim analyses are not planned. A data safety monitoring board will not be involved. A detailed statistical analyses plan will be prepared and finalized before the code is broken. Data will be analyzed according to the CONSORT (*Consolidated Standards of Reporting Trials)* statement [55]. Statistical analyses will be carried out with an established statistical software such as R (R Foundation for Statistical Computing) or SAS (Cary, NC: SAS Institute Inc.). The final dataset is only accessible for the researchers conducting the analysis at the Department of Medical Biometry and Epidemiology at University Medical Center Hamburg-Eppendorf.

### Ethics and dissemination

Ethical approval has been obtained on January 2, 2020, from the Local Psychological Ethical Commission (LPEK) at the Centre of Psychosocial Medicine at University Medical Center Hamburg-Eppendorf (LPEK-0096).

Results will be published in peer-reviewed journals and at research conferences. Important protocol modifications will be reported at the trial registry ClinicalTrials.gov.

## Discussion

PS work plays an important role in present and future psychiatric health care and can improve psychiatric treatments by its specific characteristics. The aim of this study is to investigate the effects of PS work within the HT setting, e.g., as PS workers might act as door-openers and facilitate access to treatment for patients with SMI and add valuable components to standard care by professionals without personal experiences in mental crises. Important aspects of PS work are building a trustful relationship based on shared experiences, focusing on recovery as PS workers are acting as role models for recovery and symptom management, translating between care givers and patients, and helping in coping with stigmatization of SMI in society [56]. Regarding the little research on PS involvement in HT, to our knowledge, there is only one Australian study that evaluated a combination of HT and PS – a PS service providing early discharge and hospital avoidance support for consumers with mental health conditions (*n* = 49). So-called hospital avoidance packages included home visits and met the criteria of HT [44]. During the first three months, more than 300 bed days were avoided through the PS service [57].

While the majority of HT users have a SMI and both HT and PS are explicitly recommended for them [38], according to a recent study from Switzerland on HT [58], patients who show a lower symptom severity and are still employed benefit the most from PS, which might broaden HT’s target population.

On the one hand, the key limitation of this study might be the differences in local realization of HT and PS because each participating study site has its own tradition and conditions in both treatment approaches. On the other hand, this fact reflects the reality of the German psychiatric landscape and thus ensures transfer into “real life”.

HT is an important alternative to inpatient treatment and PS involvement could support its efficacy and acceptance by users. To facilitate its further implementation into mental health care, there is a need for high quality research. The present study seeks to investigate the combination of both innovative approaches – HT and PS – to provide important information about its benefits and potential to improve mental health care for patients with SMI by support of recovery and prevention of hospitalizations.

## Data Availability

Study material and data will be available upon request.

## Abbreviations

HT: Home Treatment
SMI: Severe Mental Illness
PS: Peer Support
HoPe: Home Treatment with Peer Support
RCT: Randomized Controlled Trial
CRT: Crisis Resolution Teams
IEHT: Inpatient Equivalent Home Treatment
NICE: National Institute for Health and Care Excellence
EX-IN: Experienced Involvement
SWE: Generalized Self-Efficacy Scale
ISMI: Internalized Stigma of Mental Illness
CGI: Clinical Global Impression
GAF: Global Assessment of Functioning
ZUF-8: Client Satisfaction Questionnaire
ITT: Intention-to-treat
PP: Per-Protocol
AE: Adverse Events
CONSORT: Consolidated Standards of Reporting Trials
LPEK: Local Psychological Ethical Commission
GDPR: European General Data Protection Regulation
BDSG: German Federal Data Protection
SGB: Security Law Book
